# Wastewater Surveillance: A National Concept for Germany—A Refined Approach to Surveillance Site Selection

**DOI:** 10.3390/microorganisms14061197

**Published:** 2026-05-26

**Authors:** Thomas Exner, Ines Flügel, Timo Greiner, Marcus Lukas, Nathan Obermaier, Peter Pütz, Cristina J. Saravia, Alexander Schattschneider, Antje Ullrich, Ulrike Braun

**Affiliations:** 1 German Environment Agency (UBA), Wörlitzer Platz 1, 06844 Dessau-Roßlau, Germany; thomas.exner@uba.de (T.E.); marcus.lukas@uba.de (M.L.); nathan.obermaier@uba.de (N.O.); antje.ullrich@uba.de (A.U.); 2 Robert Koch-Institute (RKI), Seestraße 10, 13353 Berlin, Germany; greinert@rki.de (T.G.); schattschneidera@rki.de (A.S.)

**Keywords:** public health, wastewater surveillance, wastewater-based epidemiology, sampling strategy, SARS-CoV-2, COVID-19

## Abstract

During the coronavirus disease-2019 (COVID-19) pandemic, wastewater-based surveillance (WBS) gained renewed importance by enabling nationwide assessment of severe acute respiratory syndrome coronavirus 2 (SARS-CoV-2) circulation independent of individual testing. In Germany, WBS was established by a series of initiatives, including the AMELAG project, established in 2022. As the pandemic phase waned, the extensive surveillance infrastructure—comprising around 170 wastewater treatment plants (WWTPs)—was scaled down. A subset of 53 WWTPs was selected by a hierarchical set of criteria to ensure continuation of WBS in 2025, resulting in a reduced population coverage (26% instead of 37%), preserving the monitoring of infection dynamics while improving operational efficiency. The multi-stage selection approach integrated operational experience and performance data of WWTPs collected between November 2022 and July 2024, including wastewater characteristics and laboratory quantification success, minimum population coverage across all administrative regional states of the country, and statistical quality metrics such as the frequency of outliers and implausible inflexion points or the deviation from LOESS regression trends. Additional consideration was given to sites of extended research relevance. Reducing the number of WWTPs by more than two-thirds did not result in notable deviations in the aggregated national SARS-CoV-2 viral load profile. However, the evaluation was limited to SARS-CoV-2 data, despite ongoing expansion of the WBS network to include additional pathogens. Overall, the data-driven site-selection framework, developed from scientific and operational criteria, ensures the sustainable continuation of the nationwide WBS system.

## 1. Introduction

Wastewater represents a valuable source of health-related information on a wide variety of pathogens, antimicrobial resistances (AMRs), patterns of licit and illicit substance use, and the consumption of pharmaceutical agents [1]. Wastewater-based surveillance (WBS) aims to leverage this information, notably to monitor the spread of infectious diseases by detecting pathogens excreted by infected individuals into the sewage system. This approach allows the detection of rare pathogens, e.g., poliovirus, and new emerging threats as an early detection system as well as allowing the monitoring of infection dynamics of common pathogens, e.g., severe acute respiratory syndrome coronavirus 2 (SARS-CoV-2) and influenza viruses [2]. During the coronavirus disease-2019 (COVID-19) pandemic, surveillance of SARS-CoV-2 and its variants emerged as a central focus of WBS efforts worldwide, leading to a significant increase in scientific research on WBS [3]. Since then, WBS has proven to be a valuable complementary tool for assessing pathogen infection dynamics [1,4,5] in a practical and economically feasible manner [6]. The strengths of WBS are (I) the independence of clinical testing strategies and the populations testing behaviour, (II) the coverage of large populations, including asymptomatic cases, and (III) advantages regarding individual data protection as the samples represent the catchment area of a wastewater treatment plant (WWTP) [7], which can be considered ethical benefits of a surveillance system regarding privacy standards [8]. However, WBS has clear limitations, e.g., it is not possible to draw conclusions about disease severity from wastewater data, individual infection control measures cannot be derived, and external factors such as rainfall can heavily affect the data [5,9,10]. Furthermore, reducing the complexity of networks can provide an additional improvement, since funding of surveillance systems can be challenging in some countries [11,12]. According to the World Health Organisation (WHO) and the Global Consortium for Wastewater and Environmental Surveillance for Public Health (GLOWACON) operational feasibility and cost reduction are considered a priority in the implementation and improvement of WBS systems [10]. According to the U.S. National Academies of Sciences, Engineering, and Medicine, the temporal and spatial resolution of WBS programmes should be intentionally designed and continuously refined through rigorous and iterative analysis of data for prioritised pathogens [4]. Geographic, demographic, and socioeconomic considerations were further taken into account for site-selection processes [13].

In Germany, about 96% of the population in 2021 was connected to a municipal WWTP according to the German Federal Statistical Office [14]. In 2022, a total of 8.33 billion cubic metres of wastewater were treated by 8659 municipal WWTPs in Germany, representing 115 L per population equivalent (p.e.) and per day [15]. One p.e. represents the organic biodegradable load of one person or other source, e.g., industrial discharge, per time, usually taken as 60 g of a five-day biochemical oxygen demand (BOD_5_) in one day [16]. Approximately 22% of Germany’s WWTPs have a size of more than 10,000 p.e. and treat about 90% of the domestic wastewater in Germany [17]. In Germany, the relation between urban and rural areas is often defined by functional criteria regarding population distribution, which indicate higher population growth trends in urban areas as well as a growing connection between suburban areas and larger metropolitan regions, often referred to as ‘BIK’ [18,19]. In terms of administration, Germany has a federal structure, consisting of 16 federal states (Bundesländer), each with its own government and considerable autonomy in areas such as education, law enforcement, and cultural affairs, which influences governance and regulation of the wastewater sector [20]. The total population of the Federal Republic of Germany is estimated to be around 84 million in 2024 [21].

The implementation of WBS in Germany can be described by a series of projects. Early in the pandemic, the joint research project CoroMoni was initiated and funded by the Ministry of Education and Research (BMBF) with one sequencing location and a total of 24 WWTPs in three federal states for regular analysis, while other local research projects funded an additional 13 sampling sites.

In March 2021, the European Commission issued a request for the EU member states to establish nationwide, systematic WBS of SARS-CoV-2 and its variants (EU/2021/472) [22], which led to the pilot project ESI-CorA (Emergency Support Instrument—Detection of SARS-CoV-2 in Wastewater) [23]. A primary objective of this project was to assess the feasibility and methodological requirements for reliable SARS-CoV-2 WBS. The selection of WWTPs was based on criteria that allowed a distribution among all federal states, involving large and small WWTPs, and considered the integration of regions with tourism activity. For data analysis, WWTPs from the CoroMoni project were also included. To ensure quality across the workflow—from sampling to analysis—harmonised protocols for the WWTPs and for the involved laboratories were provided. In brief, samples from around 50 WWTPs—with at least 1 WWTP per federal state despite an uneven distribution—were taken two times a week and analysed for specific SARS-CoV-2 gene fragments in one of thirteen participating laboratories. Variant sequencing was performed three times at all WWTPs simultaneously during the runtime of the project. Overall, ESI-CorA established the first nationwide WBS infrastructure for SARS-CoV-2, with the potential for further expansion and improvement.

The national project about wastewater monitoring for the epidemiological situation assessment (AMELAG) was initiated in November 2022 by the German Ministry of Health (BMG) and was jointly coordinated by the two federal authorities, Robert Koch Institute (RKI) and the Federal Environment Agency (UBA). In the framework of the AMELAG project, the SARS-CoV-2 WBS network expanded to a total of 168 participating WWTPs and 2 additional canal sampling sites, including those WWTPs that already participated in CoroMoni and ESI CorA. For routine analysis, 24 h composite samples were taken twice a week for the monitoring of SARS-CoV-2 infection dynamics by automatic sampling devices. Furthermore, eight locations, mostly WWTPs and one airport site, were sampled weekly for SARS-CoV-2 variant sequencing analyses. Since 2024, the scope in AMELAG expanded to include WBS of influenza viruses and the respiratory syncytial virus (RSV) [2]. An overview of all involved WWTP sites is given in the Supplementary Material.

The site selection in AMELAG was primarily based on criteria defined by the individual German federal states. These criteria included geographic distribution and population density related to the size and scale of each of the WWTPs, technical and operative requirements, and the voluntary participation of WWTPs. Monitoring and master data were recorded via a central web application (https://app.pia-monitor.de/) hosted by UBA to store and process data from both WWTPs and laboratories.

While comparability and validation of various polymerase chain reaction (PCR) detection technologies, laboratory workflows and target enrichment methods were addressed in the ESI-CorA project [23], AMELAG focused on harmonisation by guidelines and interlaboratory comparison approaches to enhance the understanding of comparable laboratory analysis and its limitations [24,25]. Between 2022 and 2024, a total of 22 laboratories participated in the programme in a decentralised structure. These laboratories included commercial laboratories, state and federal laboratories, and university laboratories. The workflows of the individual laboratories differed, as the guidelines allowed the laboratories a certain amount of leeway in performing the analyses. As a result, the laboratories used different methods for enriching and extracting viral nucleic acids as well as different PCR detection methods. The different workflows were compared in a complex interlaboratory test in 2024 and the results are currently still under evaluation. The first phase of AMELAG ended in December 2024, with continuation intended. Beginning in January 2025, the programme transitioned directly into its next phase, under revised conditions. A site-selection mechanism was required for a reduced approach.

As the public health emergency phase of the SARS-CoV-2 pandemic subsided, WBS systems faced increasing economic and operational challenges correlating with reduced funding, complex management due to a high number of participants and the transition toward sustainable multi-pathogen surveillance [13,16,26]. Reducing the number of sampling sites and participating WWTPs was identified as a key strategy for optimisation of the cost–benefit ratio, i.e., to lower operational costs while maintaining reliable and representative national surveillance coverage and pandemic preparedness [27].

A principal aim of the revised site-selection concept in AMELAG was to establish a reduced yet strategically representative network of WWTPs to sustain WBS from 2025 onward, while achieving operational efficiency and maintaining its monitoring capability. To realise these aims with a smaller number of WWTPs, a methodical, science-based approach was essential. Quantifiable selection criteria were identified and a ranking system applied.

The primary selection criterion was population coverage, as it was a key factor for generating large data sets that enable the detection of novel pathogens and provide insights into infection dynamics across a substantial portion of the population, while limiting the number of WWTPs.

A secondary objective was to maintain stability in population coverage across demographic regions. Thus, criteria were established that addressed the different administrative subsections of Germany by setting coverage benchmarks relative to the population of each federal state and to ensure fair political representation and inclusion of all regions. Since Germany is a federal country, at least two WWTPs from each of the sixteen federal states were included, where feasible, to ensure adequate political and administrative representation; however, in two of the three city-states, only a single plant was taking part in AMELAG, while one of them offers two distinct inlets as sampling sites. An additional goal was the inclusion of all metropolitan and commuter regions, but this might not cover all cross federal-state or city-state boundaries. While the surveillance of transportation hotspots such as airport sampling [28] is also an important part of Germany’s overall WBS strategy, it is not considered part of the scope of the following selection process. Additional WWTPs were considered by demands of international projects that focus on sensitive regions, traffic hotspots, and additional research interests. The European Super-Sites Sentinel System (Supersites) comprises a network of strategically located sampling sites across the EU, designed to monitor pathogens and other health indicators [29]. The sites are positioned near key transportation hubs or at other locations of interest such as hospitals. These sites are intended to provide epidemiological insights into the presence and spread of pathogens, serving as an early detection system for new health threats entering through international travel [30]. Besides WWTPs, the project includes samples from aircraft and airports, thus broadening the scope of surveillance. The project monitors multiple pathogens and includes metagenomic analyses of wastewater samples in order to support pandemic preparedness efforts of the European Commission [31]. Therefore, WWTPs participating in the Supersites were to be included in the national programme.

As a third objective, insights gained from the analysis of previous projects were considered to identify favourable WWTPs for the selection. This selection approach for the WWTPs followed statistical criteria related to data quality to improve trend analysis, confidence intervals, and outlier reduction.

Combining all these aspects, explicit site-selection criteria for providing a continuous, high-quality, nationwide epidemiological assessment for Germany were established. These “guiding criteria” ensured comprehensive population and spatial coverage as well as reliable data quality with the selected WWTPs.

## 2. Materials and Methods

### 2.1. Data Sources

Data sources for selection were historical SARS-CoV-2 viral load data from AMELAG from November 2022 to July 2024. For this purpose, data from the laboratories performing analyses and the central database in AMELAG (https://app.pia-monitor.de/) were included. To ensure good data quality in each sample, the laboratories were required to measure at least two distinct gene sequences of SARS-CoV-2, along with an additional surrogate virus (for quality control), using PCR-based methods. A geometric mean value of all SARS-CoV-2 genes analysed was formed and subsequently normalised by the corresponding mean daily flow rate passing the WWTP at the inflow [23].

The served population and nominal load of each WWTP were provided by the annual operating estimate reported to the European Environment Agency (EEA) for the year 2022 [32]. The total amount of indirect discharge related to industrial wastewater was covered by a survey data sheet to register a WWTP in the AMELAG programme and compared to the total value of nominal load reported to the EEA for the year 2022 according to the European UWWTD (EU 2024/3019) [16]. As part of the AMELAG programme, numerous WWTPs provided self-disclosure of p.e. values from industrial discharges into their plants. Since no generic data is available regarding the average impact of the industrial discharge due to its variety among different industries, production processes, and discharged substances, a dilution of domestic wastewater by one log10 stage was set as unqualified for further analysis (part of the minimum data quality criteria). A WWTP was also excluded in case of severe inhibitory effects observed in the PCR detection method based on internal control standards in participating laboratories. The inhibitory effects vary in their mechanisms and can be caused by industrial effluents, metals and chemical agents [33], but also by common substances found in domestic wastewater. Humic acids, urea, polysaccharides and bile salts are also assumed to cause inhibitory effects [34]. Inhibition can lead to false negative results, but these can often be corrected by using commercial inhibitor removal kits or by diluting the samples [35]. For a WWTP to be excluded, inhibition must occur regularly, lead to false negative results below the limit of quantification (LOQ), and be without successful inhibitor removal.

### 2.2. Selection Process

A set of selection criteria was compiled in order to execute the task of reducing the number of WWTPs. These criteria cover technical aspects, data quality parameters, population coverage and strategic research interests. Three sets of criteria were used to disqualify WWTPs from further selection: basic technical and operational criteria, minimum data quality criteria, and minimum population coverage. By contrast, two criteria determined the amount of required WWTPs per federal state: minimum population coverage and additional evaluation criteria. Further selection was decided by large population coverage differences and statistical data quality ranking criteria. The following set of selection criteria was applied:

#### 2.2.1. Basic Technical and Operational Criteria

- The WWTP participated in the AMELAG programme in the year 2024. This criterion ensured that tasks of the programme could continue seamlessly with existing technical know-how, infrastructure and that comparative data could be provided for further selection criteria.
- Technical minimum criteria were sampling requirements. This included the installation of an automatic sampling device for WWTP influent samples after an existing mechanical cleaning stage, like a grit chamber.
- Samples had to be taken in accordance with provided technical guidelines [24] related to DIN 38402-11 [36] (A11 Sampling of Wastewater) (February 2009 edition) and the DIN EN ISO 5667-16 [37] Sampling for Biological Test Procedures (March 2016 edition). In general, 24 h composite samples had to be taken by a temperature-controlled (5 ± 3 °C) samplers in accordance with DIN EN 16479 [38] on the performance requirements [24] and conformity testing of automatic samplers. A homogenised, labelled sample had to be provided. Additional information on wastewater characteristics, e.g., the daily inflow rate, water temperature, conductivity and pH value, had to be provided by the WWTP to the analysing lab.

#### 2.2.2. Minimum Data Quality Criteria

- The existing data set in AMELAG for a WWTP candidate for the selection process must contain at least 15 different measurement days on which SARS-CoV-2 concentrations were determined to be above the laboratory analytical limit of quantification (LOQ).
- No severe inhibitory effects on the PCR analyses caused by the local wastewater matrix were observed by applying internal control standards during PCR analysis.
- The proportion of industrial and commercial wastewater in the municipal WWTP had to be below 90% of the wastewater mixture. Since SARS-CoV-2 data are mostly related to the domestic wastewater of the population, contribution from industrial effluents can be suspected to cause additional stages of uncertainty in the evaluation related to the representativeness of the sample [39]. Since a multi-pathogen approach and a variety of analytical methods are used, no universal threshold of validity can be defined and a conservative cut-off of one log10 step was taken.

#### 2.2.3. Minimum Population Coverage Criteria

- The minimum number of WWTPs to be included was set at two WWTPs per federal state. This rule may not fully apply to the city-states with less than two participating WWTPs, e.g., Bremen and Hamburg. This criterion provides a reference value for each region, which can be seen useful in case of unplanned incidents, system outages, or process delays.
- The selected WWTPs per federal state should cover, in total, at least 15% of its population. This benchmark value relative to the regional population aims to ensure a fair distribution across different regions in Germany. This criterion could be waived for federal states with a low urban population density and a low population coverage by all participating WWTPs of the region, e.g., Brandenburg. The 15% minimum coverage level emerged from a political and economic stakeholder process.
- Smaller WWTPs (state population coverage ≤ 2%) were only included if 15% state coverage could not be achieved with the larger WWTPs. These smaller WWTPs were selected when they fulfilled the minimum criteria and chosen based on the next highest population coverage. This criterion was applied to reduce the number of participating WWTPs by excluding sites with limited relevance for population coverage. The cut-off value was pragmatically defined to ensure a reduction in states with a high number of participating small WWTPs to adjust the sampling strategy more balanced between the federal states of Germany related to the number of WWTPs.
- The size of the WWTP according to its nominal load could be an additional criterion in case a tie-breaker was needed. This could be particularly relevant if a WWTP serving more than 1% of Germany’s population was competing against a WWTP serving a smaller population, or if the larger WWTP outperformed the smaller WWTP in the same federal state by more than twofold in the selection process.

#### 2.2.4. Additional Evaluation Criteria Related to Research Interests

Additional WWTPs were selected within a federal state to improve the local reference data and the overall data quality of a federal state if a participating WWTP is connected to:

- An additional research argument related to specific research projects or supplementary use of data. Examples include mobility data concepts for population coverage optimisation [40], as well as the UBA internal research database (Sampling side of the German Environmental Specimen Bank in Dessau).
- The first announced EU super-sites in Germany, also called Supersites, are Frankfurt/M. (WWTP and FRA airport), Berlin (WWTP Waßmannsdorf and BER airport), and Munich (WWTP and MUC airport). Not all German Supersites were also part of the AMELAG project. German WWTPs that are part of both, AMELAG and the EU super-sites, were selected. This usually excludes Airport sampling. However, some EU super-sites like in Düsseldorf (WWTP and DUS airport), and in Aachen (WWTP), were announced after the selection process in this article and were therefore not considered as EU super-sites in the selection process.

#### 2.2.5. Statistical Data Quality Ranking Criteria

Three statistical selection criteria were computed separately for each WWTP, then all analysed WWTPs were ranked according to each criterion and a single score as the mean value of the three rankings was formed. The rankings were counted from one, as the most unfavourable statistic evaluation result, to the highest number, as the most favourable statistical result. The single score as mean value of the federal rankings was used as a selection criterion on a state level in order to compare different WWTPs associated with the same laboratory and analytical method connected to the related federal state. One exception was the federal state North Rhine–Westphalia, where separate analyses were carried out for the various participating laboratories. Here, the four involved laboratories were evaluated independently from each other to avoid bias related to a different laboratory workflow.

R (Version 4.5.1) or RStudio (Version 4.5.1) were used to calculate the following statistical data quality criteria:

1. Reproduction rate outliers and implausible infection points (RIIP):

The proportion of implausible points (outliers) in the SARS-CoV-2 viral load curve was calculated and evaluated as a trend quality assessment based on the reproduction rate $R_{w,t}$ according to Saravia et al. (2024) [41]. The reproduction rate ($R_{w,t}$) was derived as the nth root of the ratio between the SARS-CoV-2 viral load at time *t* (${SC}_{t}$) and that was recorded *n* days before (${SC}_{t-n}$), where n denotes the time interval between measurements in days (Equation (1)).

(1) $$R_{w,t}=\;\sqrt[{n}]{\frac{{SC}_{t}}{{SC}_{t-n}}}$$

It is assumed that the reproduction rates of WBS data are related to the estimates from clinical case data and exhibit limited variability across distinct outbreak events [42]. Markedly elevated or reduced $R_{w,t}$ values were indicative of abrupt deviations in the concentration trajectory, whereas smoother transitions were interpreted as rather consistent trends. The distribution of $R_{w,t}$ values across all calculated WWTPs was employed as a baseline reference to identify two statistical irregularities. Outliers in $R_{w,t}$ are data points, calculated to the previous sampling point, that were more than 1.5 times the interquartile range (IQR) above the 75th percentile or below the 25th percentile of the overall $R_{w,t}$ values, where IQR is defined as the range between the 75th and 25th percentiles. A SARS-CoV-2 concentration value at time *t* was deemed implausible if the corresponding $R_{w,t}$ exceeded the 75th percentile of the reference distribution while the immediately following $R_{w,t}$ dropped below the 25th percentile or vice versa, indicating an unrealistic and abrupt change in trend direction (implausible inflexion points).

For each WWTP, the frequency of data points meeting either of the two criteria was calculated in relation to the WWTP total data points and employed as the implausible infection points (RIIP) criterion for ranking, assigning lower ranks to WWTPs exhibiting higher proportions of implausible measurements.

2. Ranking of median absolute error (MAE):

This ranking was based on the average deviation of each individual value from the WWTP-specific Locally Estimated Scatterplot Smoothing (LOESS) regression curve. LOESS is a form of moving average methodology used for data smoothing. It facilitates the visualisation of underlying patterns within the data by reducing the influence of outliers and high-frequency noise and can, therefore, be applied to evaluate the variability of WBS data sets [43]. To assess the variability of SARS-CoV-2 viral loads around their corresponding smoothed curve, the median absolute error (MAE) was calculated for each WWTP data set based on the deviation of observed values from the LOESS regression curve. The observed SARS-CoV-2 concentrations were log10-transformed prior to the formation of the LOESS function and the MAE calculation. This logarithmic transformation enabled interpretation of deviations in relative terms since the difference between two logarithmic values (log10(x) − log10(y)) is equivalent to the logarithm of their ratio (log10(x/y)). Consequently, the absolute errors reflect the distance between measured and modelled values as a relation, thus reducing the influence of scale differences across data sets. The resulting log-scale MAE values served as a standardised metric for evaluating the stability and thus quality of the time series data. These MAE values were subsequently used to rank data sets, with lower MAE values achieving higher scores, indicating a better fit to the LOESS trend and, by extension, a higher data quality under consistent analytical conditions.

3. Ranking of analytical quantification success (above LOQ values):

The limit of quantification (LOQ) represents the lowest analyte concentration (here SARS-CoV-2 genes) that can be reliably quantified with acceptable precision and accuracy under defined experimental conditions. Measurements below the LOQ threshold are subject to disproportionately high relative uncertainty and potential bias, which can compromise the validity of quantitative analyses and subsequent interpretations for trend analysis. For this reason, values below the LOQ have been excluded for quantitative evaluation but have instead been reported as “below LOQ value”. For each WWTP, the proportion of measured data points below the LOQ has been ranked in a comparable time frame, with the highest proportion achieving the lowest rank. Ties in the ranking were given the same number, which is why the highest value does not have to correspond to the total number of WWTPs. Since some areas may have fewer cases of SARS-CoV-2, and LOQ limits vary among participating laboratories due to differences in analytical methods, expected caution, and confidence, this criterion has limitations and cannot replace a solid data quality evaluation. However, it can attribute to it. It is assumed that sites with a high frequency of values above the LOQ are more likely to provide sufficient genomic material for analysis. By contrast, low population density, matrix inhibition, and advanced degradation of viral RNA within the sewer system may compromise data quality.

### 2.3. National Trend Analysis

The impact of the overall reduction can be illustrated using a national trend curve based on the aggregated viral load and its temporal development in Germany. By computing such a curve for both all WWTPs and only the selected WWTPs, the overall shape as well as the timing of peaks and troughs can be compared. To compute this curve, the data are processed into weekly mean viral load values and adjusted for systematic differences across site–laboratory combinations before being aggregated across all WWTP monitoring sites. Weighting is performed according to the population served by each WWTP [2,19,41]. The national trend curve was then estimated using LOESS regression based on the aggregated data. As the amount of data available from participating WWTPs and successful measurement results can vary between time points, an additional weighting step accounts for differences in uncertainty of the regression curve by assigning lower weights to mean values with greater uncertainty [44]. The LOESS curve is presented together with pointwise 95% confidence bands (constructed using the corresponding t-distribution quantile). National WBS trends for SARS-CoV-2 generated by the described LOESS regression are published weekly by the RKI in Germany [2,24].

## 3. Results

### 3.1. WWTP Selection Results

**Figure 1:**
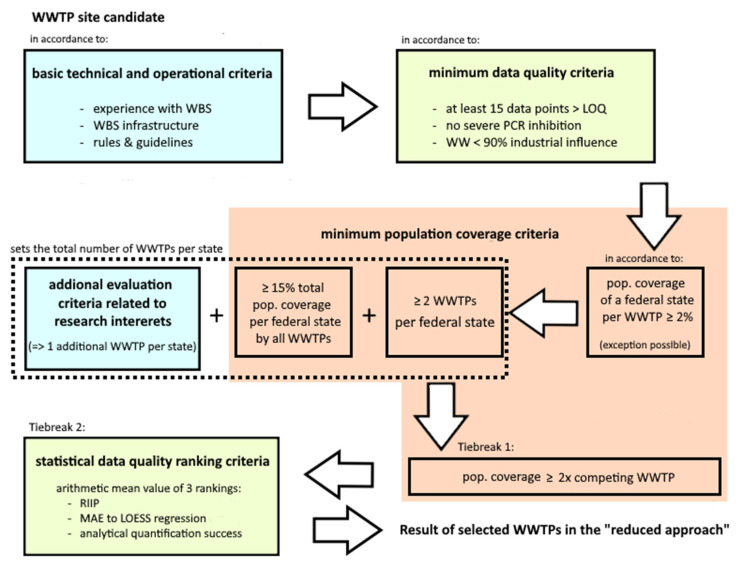
Flow chart with hierarchical order of criteria in the selection process to reduce the number of WWTPs in Germany’s WBS network. Blue criteria represent organisation, engagement, and the general or strategic qualifications of a participating WWTP. Green criteria refer to the provided data, the data evaluation process, and the results. Red criteria are related to population coverage at regional and federal levels.

The selection criteria were applied in a defined order to operationalise the guiding principles and to reduce the number of WWTP sampling sites (see Figure 1). The subsequent selection stages considered the following dimensions: the basic technical requirements and officially stated research priorities of a WWTP (blue); data availability; and the historical performance of PCR measurement results at the individual WWTP sampling sites during SARS-CoV-2 monitoring in the first AMELAG period (green). Population coverage and political requirements to ensure federal balance were also considered (red).

Through a structured, multi-stage selection process, the number of WWTPs was reduced to 53 for the year 2025 (see Table 1). The order went from a checking of the site’s basic technical and operational criteria to the ability to process data that met the minimum quality criteria required for further evaluation related to the exclusion of certain WWTPs. The latter included the proportion of industrial discharge, the observation of severe inhibitory effects and the overall number of available measurement points. Three different population coverage criteria were then applied. A minimum of two treatment plants per federal state had to be selected, if possible, to cover all federal administrative sections of Germany. In addition, a minimum population coverage of 15% of all federal states had to be achieved. In this selection process, WWTPs serving at least 2% of their local federal state population were prioritised. Additional strategic research interests in a region led to the possibility of funding one more WWTP surveillance site than was needed to achieve 15% population coverage of a federal state. A ranking of the potential data quality of a site, related to the evaluation of previous SARS-CoV-2 measurements (by RIIP value, MAE value, and the relative number of measurements above LOQ), and an overall higher population coverage than that of the next possible WWTP candidate in the same federal state (at least twice the coverage) were used as a tie-breaker, with preference given to the latter.

Of the original 170 AMELAG sampling sites, 168 fulfilled the baseline technical and operational requirements and were thus eligible for further evaluation. Excluded were two canal sites, which were not WWTP sites. Among these 168, 37 sites were disqualified based on the minimum data quality criteria. The exclusion was based on high levels of industrial discharge, observed inhibitory effects, or the lack of data related to an insufficient number of measurement results above the LOQ. Among them, the exclusion due to high loads of industrial discharge was dominating.

In the initial filtering step of the population coverage criteria, the number of WWTPs was reduced to 65 based on a minimum threshold of 2% population coverage per federal state. However, some states—such as Baden–Württemberg and Rhineland–Palatinate—retained WWTPs below this threshold to satisfy additional selection criteria. The final allocation of WWTPs per federal state was determined by achieving a minimum of 15% coverage of the state’s total population served by WWTPs, as well as by ensuring representation from at least two different cities and WWTPs per state (with exceptions for city-states). In cases where intensified official research interest was declared for a specific site or region, an additional WWTP was considered for inclusion. About half of the federal states provided an additional research interest.

**Figure 2:**
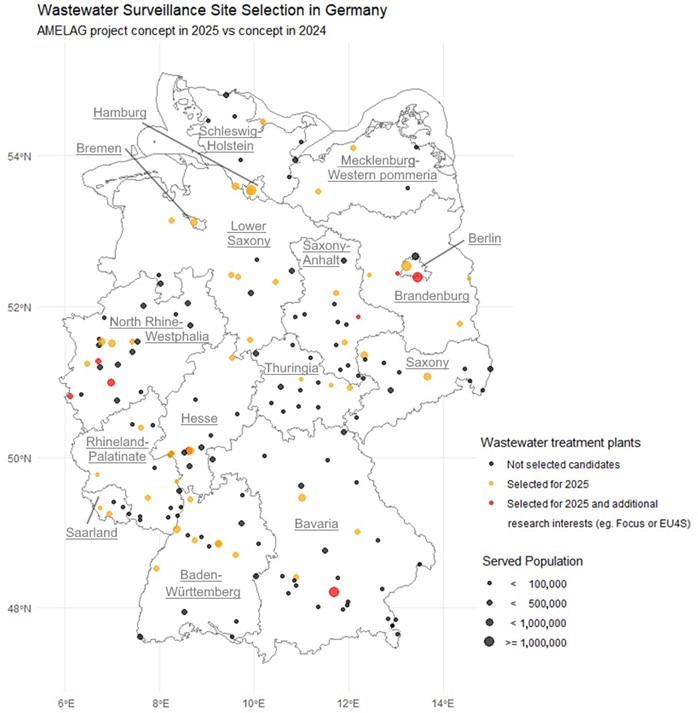
Wastewater Surveillance Site Selection in Germany—AMELAG concept in 2025 vs. concept in 2024. Dots indicate participating WWTPs, with colours illustrating the respective stage of the selection process. Dot size reflects four categories of population served, representing the capacity or catchment size of a WWTP.

Tie-break decisions between candidate sites were resolved based on either superior population coverage or, alternatively, higher performance in statistical data quality comparison results. Finally, 53 WWTPs were identified as suitable for the scaled-down approach for the amount of WWTPs in AMELAG. The distribution across Germany and the WWTPs’ capacities (in served population) are illustrated in Figure 2. More detailed information can be found in the Supplementary Material. The direct population coverage of Germany via WBS can be estimated above 26% of the total population in 2025 instead of above 37% in 2024. These values do not consider travel and commuting behaviour.

The results of the inclusion criteria and WWTP selection across the German Federal States are shown in Table 1 and summarised as follows:

- In Baden–Wuerttemberg, the population coverage criterion was central. Only three WWTPs exceeded the 2% population coverage at the state level, while four additional WWTPs had to be included to meet 15% total coverage.
- In Bavaria, four of the twenty-seven WWTPs were selected for their substantial population coverage and data quality. While only three WWTPs were needed to fulfil the 15% coverage criterion for Bavaria, all four WWTPs were chosen due to the additional evaluation criterion related to Munich taking part in the Supersites.
- In Berlin, two major WWTPs met all criteria. While the rule requiring two WWTPs per state does not apply to city-states, both WWTPs were still included because of the additional research interest rule, since the WWTP Berlin–Waßmannsdorf is an EU super-site.
- In Brandenburg, all four WWTPs were considered and did not achieve the 15% total coverage goal. However, large WWTPs associated with Berlin in this concept also cover parts of Brandenburg, which could compensate for the missing coverage.
- In Bremen, the only participating WWTP (86% coverage) was selected, mostly covering the city of Bremen itself and not the city of Bremerhaven.
- Both sampling sites in Hamburg belong to the same WWTP with separate inflow streams. Therefore, both sites were taken, counting as one WWTP.
- In Hesse, four sites were selected, including Frankfurt/M. (EU super-site) with two sampling sites as well as Wiesbaden and Kassel, which were chosen based on the data quality ranking criterion.
- In Lower Saxony, the population coverage criterion led to the clear result that all five WWTPs stayed in the programme.
- In Mecklenburg–Western Pomerania, two WWTPs that achieved the highest values in population coverage and in the statistical ranking criterion were selected.
- The selection for North Rhine–Westphalia retained five WWTPs. While only four of them were required to meet the 15% population coverage criterion, the additional evaluation criterion led to one more site, since Cologne is a focus site in AMELAG. Two additional EU super-sites were announced in summer 2025 in North Rhine–Westphalia, after the described selection process, leading to additional financed WWTPs in 2025 as surveillance sites that are not part of this analysis.
- For Rhineland–Palatinate, five of the sixteen AMELAG WWTPs were selected based on population coverage necessities.
- While in Saarland, one WWTP was fixed to be selected due to its large population coverage and high data quality, a second candidate was chosen due to the data quality ranking criterion.
- In Saxony, two participating WWTPs outperformed all other WWTPs by population coverage and size significantly (more than 2 times). Therefore, the population coverage tie-break criterion was crucial for the decision.
- In Saxony–Anhalt, two WWTPs had more than twice the population coverage compared to other potential WWTP candidates. In addition, a third site was taken due to proximity to national research interests near the Headquarters of the Federal Environment Administration.
- In Schleswig–Holstein, a large WWTP (18% coverage) was clearly preferred based on the size criterion, while the second WWTP was selected based on the statistical data quality ranking criterion.
- In Thuringia, three WWTPs were selected, one due to exceptional population coverage, and two due to statistical performance and research integration with relevance to artificial intelligence-based mobility studies [39] for alternative WWTP selection concepts.

### 3.2. National Trend Analysis Comparison

**Figure 3:**
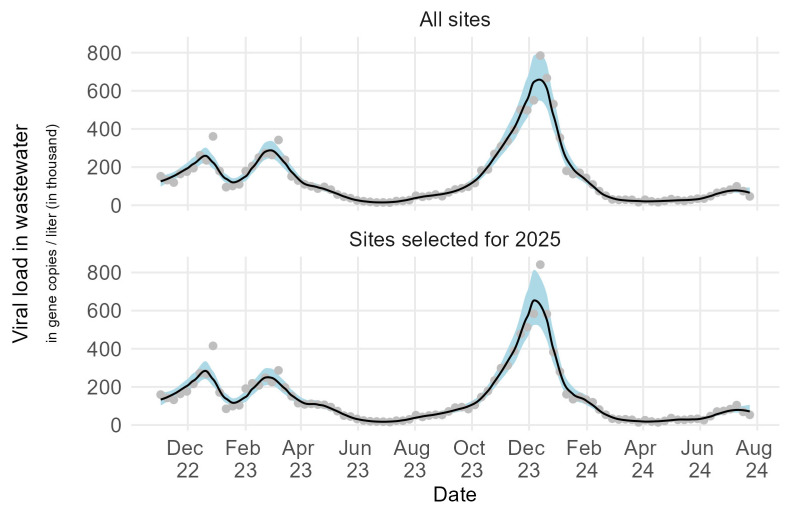
Comparison of aggregated SARS-CoV-2 viral load for all WWTPs (upper curve) vs. selected WWTPs (lower curve). Aggregated SARS-CoV-2 load in wastewater over time (grey dots) is shown along with a regression curve (black line, estimated using the LOESS method) and associated 95% confidence intervals (light blue area).

In the result, a national trend analysis of the ‘reduced approach’ with 53 WWTPs can be compared to the original 170 sampling sites of the AMELAG project. To generate a comprehensive overview of national viral load dynamics, measurements from all monitoring locations were aggregated to generate a LOESS regression curve with 95% confidence bands (Figure 3): The original curve using all sampling sites is very similar to the reconstruction with the reduced data set (Pearson’s correlation coefficient and Lin’s concordance correlation coefficient of 99%), while the latter exhibited only slightly wider confidence bands (on average 7% wider). The three bigger waves in December 2022, February/March 2023 and December 2023, as well as the troughs in June/July 2023 and April 2024, can be clearly identified at very similar time points (the corresponding local maxima and minima of the curves are shifted on average by two days). Thus, using the described selection criteria, the reduction in the number of treatment plants by about two-thirds did not lead to a notable difference in the aggregated representation of the SARS-CoV-2 viral load.

## 4. Discussion

The results of this study demonstrate an effective WBS site-selection model for Germany, leading to a scaled-down surveillance approach. The reduction in the number of regularly sampled WWTPs from 168 to 53 reflects the epidemiological transition of COVID-19 from a pandemic to endemic levels within a large WBS network. By refining the selection criteria based on population coverage, statistical data quality indicators, distribution among administrative subsections (federal states), and research relevance, the approach has successfully streamlined the number of WWTPs involved in the German WBS network, while maintaining the representativeness of national and regional epidemiological trends in an efficient manner. Key tasks such as logistics, laboratory operations, IT infrastructure and data transmission, as well as data analysis and regular communication via online platforms can continue to function efficiently within this new framework.

Operational costs of WBS are largely determined by the number of participating WWTPs [27]. Accordingly, the reduction in participating sampling sites resulted in an approximately threefold decrease in operational costs and significantly reduced need for management capacity. At the same time, nationwide population coverage decreased from 37% in 2024 to 26%, remaining within the range reported for other European WBS systems (25% to 99%) [45]. However, more extensive reductions in network density may compromise analytical robustness and confidence [27]. Despite the reduced network size, both Pearson’s correlation coefficient and Lin’s concordance correlation coefficient remained close to 0.99, indicating no substantial deviation in national trend analysis.

One key limitation of the statistical data quality ranking methodology lies in its current dependency on SARS-CoV-2 data. Current concepts require a greater focus on the extended analysis of new pathogens and antimicrobial resistance (AMR) [26,29] related to the potential need for fewer but more specialised WWTP sampling sites in Germany. During the evaluation period, insufficient processed data were available for other pathogens, such as RSV, influenza viruses, and markers relevant for AMR. Consequently, the ranking-based selection of WWTPs reflects past surveillance performance for a single pathogen and does not yet account for the broader range of indicators relevant to the evolving scope of WBS.

It can be concluded that population coverage was the dominant criterion in the selection process across Germany, followed by exclusions due to high proportions of industrial wastewater. By contrast, the influence of the statistical data quality ranking criterion was limited, playing a decisive role in only four out of sixteen federal states compared to population coverage aspects. Nevertheless, the data quality ranking improved the possibility of comparing WWTPs directly.

Germany’s federal structure has a broad geographic distribution of wastewater surveillance across the country. Unlike algorithm-driven models that optimise cost reduction in the context of avertable infection burden [46] or spatial distribution and population coverage [47], the German framework is grounded in predefined, criteria-based selection, similar to the approach in France to cover administrative subsections in addition to population coverage [12]. Criteria-based approaches often employ inclusion rationales that vary across national contexts. For example, Yeager et al. applied fixed benchmarks, including a minimum population served per WWTP and the exclusion of wastewater streams with a majority (>50%) of commercial or industrial origin [48]. In contrast, our approach adopted more moderate, context-specific criteria tailored to the heterogeneity of the German federal states and wastewater infrastructure. In the selection process, the population coverage of at least 15% of each federal state was set to maintain regional surveillance goals, while the reconstruction of the national surveillance trend was tested and successfully achieved. To ensure robust and comprehensive data sets for each federal state, a minimum standard of two WWTPs per state was set, despite literature on cost reduction advising to sample one WWTP twice rather than two different ones [46]. One main advantage of focusing on admirative subsections of Germany rather than geographic distance modelling is the reflection of equal representation of governance and structural differences between regions. This approach puts emphasis on including different levels of governance and stakeholder representation. Furthermore, ethical guidance of fair allocation and equity of surveillance systems should not be primarily based on spatial distribution [49]. For example, socio-economic differences can be measured between eastern and western federal states of Germany [50]. Therefore, ensuring an equal representation of administrative subsections can be considered an equity aspect in the German context. However, large urban WWTPs with high population coverage show a disproportionate influence on determining the national trend in the ‘reduced approach’. As population coverage was one of the main selection criteria for the reduced WWTP location concept, the average size per WWTP (expansion size) shifted from 295,000 to 603,000 p.e. As a trade-off, less information is currently available for regions with low population density such as rural areas. The lack of data from rural regions is a common problem for large WBS networks [17,51,52]. However, there is a strong connection between commuters and larger metropolitan areas in Germany, and population growth is mostly expected in bigger cities [18,19]. These cities usually have access to key transport locations and are therefore considered important for infection dynamics [24,37]. Nonetheless, in federal states with lower population density, WWTPs serving fewer than 100,000 p.e. are still sampled to achieve the overall population coverage targets. Structural differences between urban and rural federal states have had a significant influence on site composition. City-states, by necessity, are represented by large-scale WWTPs with high population coverage, whereas federal states with lower population density—particularly those with fewer metropolitan areas—are predominantly represented by medium-sized and smaller facilities. Furthermore, additional limitations are based on the limited information available about the served population. Therefore, commuting behaviour and population structure are not included in the approach. Regarding the ethics of surveillance systems, like many WBS approaches, it generates anonymous data and mirrors the whole society of the corresponding served population. This advantage also shows limitations with regard to the evaluation of specific groups like age groups, gender aspects, migration or marginalised communities in terms of equity aspects [49]. With the inclusion of focus sites and the European sentinel system via Supersites, the broader sensitivity to epidemiological surveillance and key transportation hubs was indirectly taken into account. The selection process represents an idealised snapshot of the situation at the time of assessment. Following the selection, several WWTPs had to be withdrawn and replaced (e.g., in Saarland) due to organisational constraints or insufficient staffing capacity at the facilities. In addition, some EU Super-Site projects were announced after the selection process, which subsequently resulted in additional WWTPs being funded in North Rhine–Westphalia. Furthermore, several federal states later provided data on additional WWTPs that were not financed at the federal level.

The selection is not a static process: it integrates empirical findings from past surveillance efforts, particularly with regard to data quality and consistency. This inclusion of past performance metrics allows for more informed and adaptive site selection, ensuring that chosen WWTPs do not only meet structural and demographic criteria but also demonstrate reliable analytical performance (including sampling and detection). As such, the approach balances methodological aspects with evidence-based refinement, enhancing the robustness of the national surveillance system over time and may be adaptive to further research tasks or the distribution among administrative subsections (here federal states).

By contrast, focusing more on the data quality ranking indicators as a tie-breaker between WWTP candidates might have shifted the results, leading to more WWTPs being selected while compromising overall population coverage. For example, prioritising data quality criteria over coverage tie-breaker rules in the selection process in Schleswig–Holstein would result in two smaller WWTPs being selected based on data quality performance indicators instead of one larger WWTP. Similarly, some federal states (e.g., Saxony-Anhalt) might have replaced one WWTP with an increased preference for data quality, resulting in lower overall population coverage. In Berlin, participation in the EU super-sites project determined the switch to a larger plant, despite a smaller WWTP achieving higher data quality indicator performance results. In some federal states, such as Mecklenburg–Western Pomerania and Saxony, both approaches produced identical results, with either tie-breaking criterion being favoured. The latter highlights a slight tendency for larger WWTPs to demonstrate higher data quality results more frequently.

The criterion of adequate frequency of measurements above the LOQ might exclude sampling locations with regard to inhibition or downstream data quality loss in the sewer system. Limitations and uncertainties of this criterion can occur, since the participation of different laboratories resulted in challenges for harmonisation, depicted, e.g., by individually determined, different levels of LOQ values for the cut-off point for low SARS-CoV-2 concentrations. This is relevant as part of the selection criteria, as some federal states, such as Baden–Wuerttemberg have data sets with many viral load values below the LOQ threshold related to the corresponding determined LOQ values of the analysing laboratory. However, this distortion was found to be irrelevant in the applied scenario as it primarily affected locations that had been excluded from the ranking due to their size. Furthermore, all WWTPs were compared at a federal state level, and in most federal states, all WWTPs were connected to the same analytical laboratory.

Differences in laboratory workflows and analytical methods can challenge results of data comparisons when several partners are involved, but should be reduced to a minimum where feasible. Efforts for international harmonisation and standardisation are already in progress via respective projects like the European EU-WISH programme (https://www.eu-wish.eu/) and via ISO standardisation.

Non-Poliovirus WBS emerged as a complementary tool to track infection dynamics during the COVID-19 pandemic and a large variety of pathogens can be identified for surveillance purposes [17,30]. As experience with the tool increased, desire for more efficient sampling arose, replacing a ‘sample-whatever-you-can’-approach. However, there might be specific outbreak situations requiring regional or even national upscaling of WBS again. With systematic WWTP distribution and location concepts, WBS can be an adaptable part of a pandemic preparedness concept. Therefore, it is crucial to maintain collaboration with public health and environmental authorities, laboratories as well as WWTPs and wastewater associations. These partners should adhere to procedures that enable rapid escalation of PCR analysis capacity—as warranted by epidemiologic signals, such as novel variant emergence or concerns of unusual outbreaks as part of pandemic preparedness.

## 5. Conclusions

As a result of a multi-stage selection process, a network of 53 WWTPs was established, representing 26% of the German population. The process refined the previous approach of 170 sampling sites and achieved a reduction by approximately two-thirds of operational costs while preserving data quality for national trend analysis with a correlation coefficient of 0.99.

The selection concept ensured a seamless continuation of WBS under redefined conditions, building upon the previous project-based surveillance framework. The chosen structure is designed to support sustained surveillance efforts for monitoring during the post-pandemic SARS-CoV-2 situation, offering a balanced framework to represent the German population through strategically selected sites based on an even distribution across federal state regions, high population coverage, wastewater characteristics, chronological operational success, and data quality checks in terms of outlier detection, implausible inflexion points in reproduction rates, and LOESS regression trend analysis. In cases of comparable suitability of several WWTPs, preference was given to either significantly larger WWTPs or those with higher data quality performance results. The approach reflects a compromise: all federal regions of Germany had to be represented in an appropriate manner, while usually larger WWTPs in each state region were chosen. However, this reduction strategy may lead to decreased representativeness and analytical sensitivity in sparsely populated rural regions.

The limitations of the statistical ranking methodology, particularly its reliance on SARS-CoV-2 data, underscore the challenge of adapting wastewater surveillance to multiple pathogens. As new targets like RSV, influenza viruses and AMR become increasingly relevant, the lack of multi-pathogen and AMR data sets limits the ability to fully assess regional infection dynamics relevant to future WWTP selection processes. Moreover, challenges in the practical implementation of the selection criteria, such as different analytical methods in participating laboratories and inconsistencies in reporting industrial wastewater shares, emphasised the importance of ongoing validation and harmonisation of all processes and steady communication with stakeholders. Collaborative efforts with public health authorities, laboratories, and wastewater associations will remain essential to adapt swiftly to emerging epidemiological signals, ensuring that the WBS system can respond effectively to future health threats.

The concept was successfully integrated into AMELAG, based on empirical findings and existing infrastructure from past surveillance efforts, enabling more reliable, evidence-based decision-making. While earlier phases prioritised broad inclusion and research-driven participation, the current approach prioritises fewer WWTPs, higher national population coverage, and federal-level coordination, ensuring effective, scalable surveillance.

## Figures and Tables

**Table 1:** Results of the selection process in Germany. The number of WWTP that passed the final selection process are highlighted in green by ferderal state.

Federal State	Qualified WWTP Sites	After Reduction By Minimum Criteria	With Further Reduction by State Population Coverage (≥2% of a State per WWTP)	Need for 15% Coverage Criteria (Fixed + Flexible)	Additional Site Criterion (Research or Sequencing) (# Part of Supersites)	Selection by 2 Times the Population Coverage of Next Potential WWTP Candidate	Selection by Statistical Ranking Necessary	Result (WWTP Sampling Sites Financed for the Reduced Approach)	Total Coverage of the Federal State (* Extended Coverage with Data Supply of External Partners)	Remarks
Baden-Württemberg	18	11	3	more required		-	-	7	18.07%	4 more selected below 2% coverage
Bavaria	27	14	4	1 + 2	+1 #	-	-	4	18.50% (* 28.15%)	2 of the 29 AMELAG sites were not WWTP sites
Berlin	3	2	2	+1	+1 #	yes	-	2	81.60% (* 98.80%)	cross coverage helps Brandenburg
Brandenburg	4	4	4	more required	(+1, not possible)	-	-	4	14.50%	cross coverage by Berlin sites achieves > 15% coverage
Bremen	1	1	1	1 + 0		-	-	1	86.00%	City-state that can be covered by 1 big WWTP
Hamburg	2	2	2	0 + 1	+1 (Both sites at the same WWTP)	-	-	2	≈99.00%	cross coverage; WWTPs cover more than Hamburg inhabitants.
Hesse	11	8	6	2 + 1	+1 #	-	yes	4	27.30%	
Lower Saxony	10	8	5	5 + 0		-	-	5	16.70% (* 18.40%)	
Mecklenburg-Western Pomerania	4	3	3	1 + 1		yes	-	2	22.70%	
North Rhine-Westphalia	21	18	5	3 + 1	+1 #	-	-	5/6	18.20% (* 22.50%)	
Rhineland-Palatinate	16	10	4	more required		-	-	5	16.20%	1 more selected blow 2% coverage
Saarland	4	4	4	1 + 1		-	yes	2	16.80%	
Saxony	11	11	4	1 + 1		yes	-	2	31.90%	
Saxony-Anhalt	13	13	6	1 + 1	+1	yes	-	3	32.10% (* 46.40%)	1 site is connected to UBA interests
Schleswig-Holstein	9	9	5	1 + 4		yes	yes	2	30.10%	
Thuringia	14	13	8	1 + 1	+1	-	yes	3	24.40%	additional research

## Data Availability

The original data presented in the study are openly available on https://github.com/peterpuetz2020/site_selection_amelag (accessed on 1 May 2026). Data analysis was done with R (Version 4.5.1).

## Supplementary Materials

The following supporting information can be downloaded at: https://www.mdpi.com/article/10.3390/microorganisms14061197/s1, Table S1: Sites in AMELAG. * former ESI-CorA-site, ** former BMBF project-site, *** site formerly funded by the federal state.

## Abbreviations

The following abbreviations are used in this manuscript:

AMELAG	Federal German WBS-project “Abwassermonitoring für die epidemiologische Lagebewertung” (engl.: wastewater monitoring for the epidemiological situation assessment)
AMR	Antimicrobial resistance
BMG	Bundesministerium für Gesundheit (engl. Federal German Ministry of Health)
BMUKN	Bundesministerium für Umwelt, Klimaschutz, Naturschutz und nukleare Sicherheit (engl. Federal German Ministry for the Environment, Climate Action, Nature Conservation and Nuclear Safety)
BOD_5_	Five-day biochemical oxygen demand
CoroMoni	Wastewater Monitoring to detect SARS-CoV-2 project in Germany by the DWA e.V. organisation. Full name (german): “Abwassermonitoring zur Bestimmung des SARS-CoV-2-Infektionsgrades der Bevölkerung und Aufbau eines flächendeckenden Frühwarnsystems—Koordination der Forschungsaktivitäten in Deutschland durch die DWA”
COVID-19	Coronavirus disease-2019
DIN	German Institute for Standardisation. German ISO member body.
EEA	European Environment Agency
EU	European Union
ESI-CorA	EU-Projekt Emergency Support Instrument—Nachweis von SARS-CoV-2 im Abwasser (compare Höckele et al. 2023 [23])
EU-WISH	Wastewater Integrated Surveillance for Public Health (EU-Project); Available online https://www.eu-wish.eu/ (accessed on 30 October 2025).
GLOWACON	Global Consortium for Wastewater and Environmental Surveillance for Public Health
IQR	Interquartile range
ISO	International Organisation for Standardisation
LOD	Limit of detection
LOQ	Limit of quantification
LOESS	Locally estimated scatterplot smoothing
MAE	Median absolute error
n	Number of measurement days
p.e.	Population equivalent
PCR	Polymerase chain reaction
RIIP	Reproduction rate outlier and implausible infection points
RKI	Robert Koch Institute (Federal German Health Institute)
RSV	Respiratory syncytial virus
Rw,t	Reproduction rate (dependent on the time)
SARS-CoV-2	Severe acute respiratory syndrome coronavirus 2
SCt	SARS-CoV-2 concentration at time t
SCt-n	SARS-CoV-2 concentration n days before time t (in days)
Supersites	The EU Super-Sites Sentinel System
t	time (in days)
UBA	Umweltbundesamt (Federal German Environment Agency)
UWWTD	Urban Waste Water Treatment Directive (Directive (EU) 2024/3019)
WBS	Wastewater-based surveillance
WHO	World Health Organisation
WWTP	Wastewater treatment plant
