# Supplementary material for: Wastewater Surveillance: A National Concept for Germany—A Refined Approach to Surveillance Site Selection
Source: Microorganisms. 2026 May 26;14(6):1197. doi: 10.3390/microorganisms14061197 (PMC13303325; doi:10.3390/microorganisms14061197)
Supplement: Supplementary file 1 [file microorganisms-14-01197-s001.zip › microorganisms-4305996-supplementary.pdf]

Supplementary Material (SI)

Wastewater Surveillance: A National Concept for Germany – A Refined Approach to Surveillance Site Selection

Thomas Exner [1], Ines Flügel [1], Timo Greiner [2], Marcus Lukas [1], Nathan Obermaier [1], Peter Pütz [2], Cristina Saravia [1], Alexander Schattschneider [2], Antje Ullrich [1] and Ulrike Braun [1]*

[1] *German Environment Agency (UBA). Wörlitzer Platz 1. 06844 Dessau-Roßlau. Germany.*

[2] *Robert Koch-Institute (RKI). Seestraße 10. 13353 Berlin. Germany.*

* *Correspondence: ulrike.braun@uba.de; Tel.: (030/89034323)*

Table A1. Sites in AMELAG. *former ESI-CorA-site, **former BMBF project-site, ***site formerly funded by the federal state

Federal State	Sites 2022-2024	Sites 2025	Population in the catchment area	Comment
Baden-Wurttemberg	Aalen		30,000	
	Bad Mergentheim		27,257	
	Berg		14,000	
	Donaueschingen		100,000	
	Eriskirch		36,000	
	Forchheim		500,000	
	Göppingen	x	300,000	
	Heidelberg**	x	161,477	Sequencing site (2022-2024)
	Karlsruhe**	x	500,000	
	Königsbach		27,239	
	Leonberg		80,000	
	Mühlacker-Lomersheim		25,311	
	Offenburg	x	120,000	
	Pforzheim	x	125,000	

	Schwäbisch Hall		100,000	
	Stuttgart*	x	600,000	
	Tübingen*	x	105,000	
	Weil am Rhein		103,000	
Bavaria	Altötting*		34,550	
	Aschaffenburg		102,806	
	Augsburg**	x	380,000	
	Bad Reichenhall**		19,480	
	Bayreuth***		88,906	
	Berchtesgaden**		25,000	
	Ebersberg**		12,500	
	Erlangen***		170,332	
	Freilassing		18,500	
	Freising**		51,617	
	Glonn**		5,300	
	Grafing**		14,900	
	Hof*		100,000	
	Ingolstadt		177,591	
	Königsbrunn		28,100	Canal site
	München**	x	1,240,000	Focus site 2025/<br>EU Super-Site
	Neu-Ulm		220,000	
	Nürnberg***	x	588,807	
	Passau***		54,000	
	Piding**		9,070	
	Regensburg***	x	279,474	
	Schwabmünchen		18,000	
	Schweinfurt**		82,500	
	Stadtbergen		13,800	Canal site
	Starnberg		65,142	

	Straubing***		58,895	
	Teisendorf**		8,300	
	Weiden***		43,754	
	Zusmarshausen		6,375	
Berlin	Ruhleben*	x	1,500,000	
	Schönerlinde		650,000	
	Waßmannsdorf	x	1,586,494	EU Super-Site
Brandenburg	Brandenburg an der Havel	x	82,881	
	Cottbus	x	150,000	
	Frankfurt an der Oder	x	65,444	
	Potsdam*	x	78,000	Focus site
Bremen	Bremen*	x	595,000	
Hamburg	Hamburg Inlet 1*	x	581,423	Sequencing site (2022-2024)
	Hamburg Inlet 2*	x	1,464,784	Sequencing site (2022-2024)
Hesse	Büdingen*		14,462	
	Darmstadt		133,633	
	Frankfurt-Niederrad	x	714,479	Sequencing site (2022-2024)/ Focus site 2025/ EU Super-Site
	Frankfurt-Sindlingen		245,495	Sequencing site (2022-2024)
	Frankfurt-Griesheim	x	588,252	
	Fulda		95,000	
	Hanau		140,000	

	Kassel	x	271,330	
	Marburg		77,933	
	Wiesbaden-Biebrich		130,229	
	Wiesbaden-Stadt	x	177,681	
Lower Saxony	Bramsche*		31,534	
	Braunschweig	x	280,000	
	Celle		71,244	
	Göttingen	x	170,000	
	Hannover-Gümmerwald	x	437,416	
	Hannover-Herrenhausen	x	303,144	
	Hildesheim		104,230	
	Oldenburg	x	170,000	
	Osnabrück		156,679	
	Wolfsburg		137,608	
Mecklenburg-Western Pomerania	Greifswald		61,963	
	Neubrandenburg		64,003	
	Rostock*	x	240,420	
	Schwerin	x	129,460	
North Rhine-Westphalia	Aachen**		206,424	EU Super-Site<br>(announced after selection)
	Bielefeld		171,564	
	Bonn*		279,186	
	Borken***		42,861	
	Bottrop**	x	732,616	
	Dinslaken*		56,812	
	Dortmund-Deusen**	x	399,425	
	Dortmund-Scharnhorst**		113,439	

	Duisburg**		242,172	
	Düsseldorf Nord***		333,122	EU Super-Site<br>(announced after selection)
	Düsseldorf Süd***		340,577	
	Emschermündung**	x	906,222	
	Eschweiler**		68,244	
	Gütersloh***		80,000	
	Hagen		187,768	
	Köln*	x	863,455	Sequencing site<br>(2022-2024)/<br>Focus site 2025
	Mönchengladbach**	x	411,656	
	Münster		241,200	
	Paderborn		154,378	
	Waldbröl***		8,912	
	Wuppertal**		317,483	
Rhineland-Palatinate	Andernach		47,086	
	Bad Kreuznach		75,000	
	Germersheim		28,318	
	Kaiserslautern	x	140,000	
	Koblenz	x	107,200	
	Landau in der Pfalz		54,708	
	Ludwigshafen		250,000	
	Mainz	x	250,000	
	Montabaur		24,900	
	Neustadt an der Weinstraße*		53,000	
	Pirmasens-Blümelstal		60,000	

	Pirmasens-Felsalbe		15,300	
	Speyer		73,215	
	Trier	x	97,927	
	Worms	x	78,400	
	Zweibrücken		36,498	
Saarland	Saarbrücken*	x	123,994	
	Saarlouis	x	43,027	
	Wellesweiler		44,905	
	Wustweiler		26,528	
Saxony	Bautzen		45,500	
	Chemnitz		246,807	
	Döbeln		21,092	
	Dresden*	x	700,000	
	Ebersbach		16,000	
	Görlitz		100,000	
	Grimma		19,800	
	Leipzig	x	606,548	
	Markkleeberg		27,393	
	Plauen		63,046	
	Zittau		76,000	
Saxony-Anhalt	Bernburg		37,000	
	Dessau	x	83,000	Additional UBA research interest
	Halberstadt		35,345	
	Halle/Saale	x	337,000	
	Köthen		36,000	
	Gerwisch-Magdeburg	x	278,000	Sequencing site<br>(2022-2024)
	Naumburg		40,000	
	Rollsdorf*		40,867	

	Schönebeck		72,000	
	Silstedt		70,000	
	Stendal		100,000	
	Weißenfels		33,600	
	Zeitz		35,431	
Schleswig-Holstein	Flensburg		115,000	
	Grömitz*		60,000	
	Hetlingen – Kreis Pinneberg	x	540,000	
	Husum		40,000	
	Kellinghusen		8,282	
	Kiel	x	351,644	
	Lübeck		245,000	
	Ratzeburg		14,894	
	Schleswig		49,743	
Thuringia	Arnstadt		49,743	
	Erfurt	x	37,475	
	Gera	x	300,000	
	Gleisdreieck		101,650	
	Ilmenau		10,000	
	Jena*	x	25,882	
	Leinetal		116,186	
	Linsenstein		70,000	
	Meuselwitz		8,543	
	Nordhausen		15,000	
	Saalfeld		53,000	
	Schmalkalden		50,000	
	Suhl		40,000	
	Weimar		60,000	
